# Hulk-Like Urine: A Case of Green Urine Caused by Flupirtine Intoxication

**DOI:** 10.7759/cureus.12333

**Published:** 2020-12-28

**Authors:** Maria Vilela, Diana Fernandes, Tatiana Salazar, Augusto Duarte

**Affiliations:** 1 Internal Medicine, Centro Hospitalar do Médio Ave, Vila Nova de Famalicão, PRT; 2 Internal Medicine, Centro Hospitalar Do Médio Ave, Vila Nova de Famalicão, PRT

**Keywords:** flupirtine, intoxication, green urine

## Abstract

Acute intoxications are common causes of admission to the Emergency Department (ED). Flupirtine is a non-opioid analgesic, originally used for acute and chronic pain. Because of several reports of severe liver toxicity, its use was limited to acute pain in 2013 by the European Medicines Agency. Although withdrawn from the European market in March 2018, there are still flupirtine tablets in many households, and most people are unaware of the hazards they might be facing.

A 58-year-old man was admitted to the ED after a suicide attempt with 1 g of flupirtine. He was lethargic and confused but presented no focal neurological deficits or other symptoms, and the rest of his clinical examination was unremarkable. His cerebral CAT and blood chemistry showed no alterations. The only remarkable feature was that he had green urine. After a careful literature search, a similar case was found caused by flupirtine intoxication. After 24 hours of vigilance in the ED, he improved his neurological status and his urine lost part of its greenish color. He was then transferred to the Psychiatric Department, where he presented a complete remission of the clinical alterations. A follow-up check-up showed no permanent deficits.

## Introduction

Flupirtine is a selective neuronal potassium channel opener, with N-methyl-D-aspartate receptor antagonist and type-A γ-aminobutyric receptor modulatory properties [1]. It was commercially used for the first time in Europe in 1984 for moderate-to-severe cases of acute pain [2]. Because of its muscle relaxant properties, it was frequently used for the treatment of orthopedic pain, migraines, and even neoplastic pain. However, due to an increase in the number of cases reporting liver toxicity after the use of flupirtine, in 2013, its use was restricted, becoming prohibited in Europe in 2018 [3,4]. 

Because this prohibition in Europe is relatively new, there are still a considerable number of households that have flupirtine tablets. For this reason, we present this case to highlight an extremely rare side effect of flupirtine, which has only been reported once to our knowledge.

## Case presentation

A 58-year-old man with a known medical history of arterial hypertension and type 2 diabetes mellitus was admitted to the Emergency Department (ED) for prostration in the last 14 hours. According to his wife, he had taken 10 100 mg tablets of flupirtine in a suicide attempt. She also reported that in the last three months he had stopped all his usual medications (clopidogrel, metformin, telmisartan, simvastatin), but denied changes in his behavior or other alterations.

**Figure 1:**
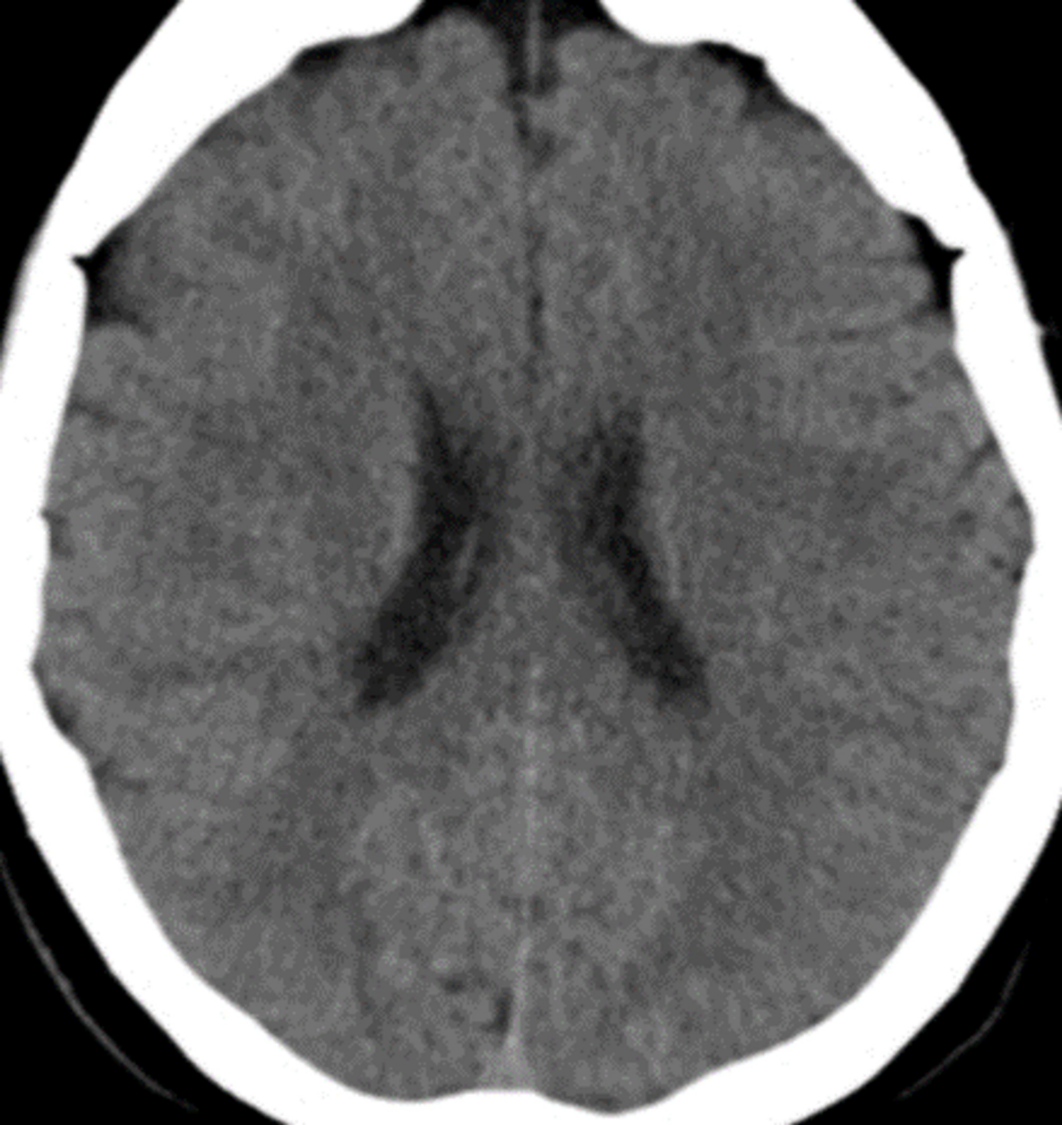
Cerebral CAT CAT, computerized axial tomography

**Figure 2:**
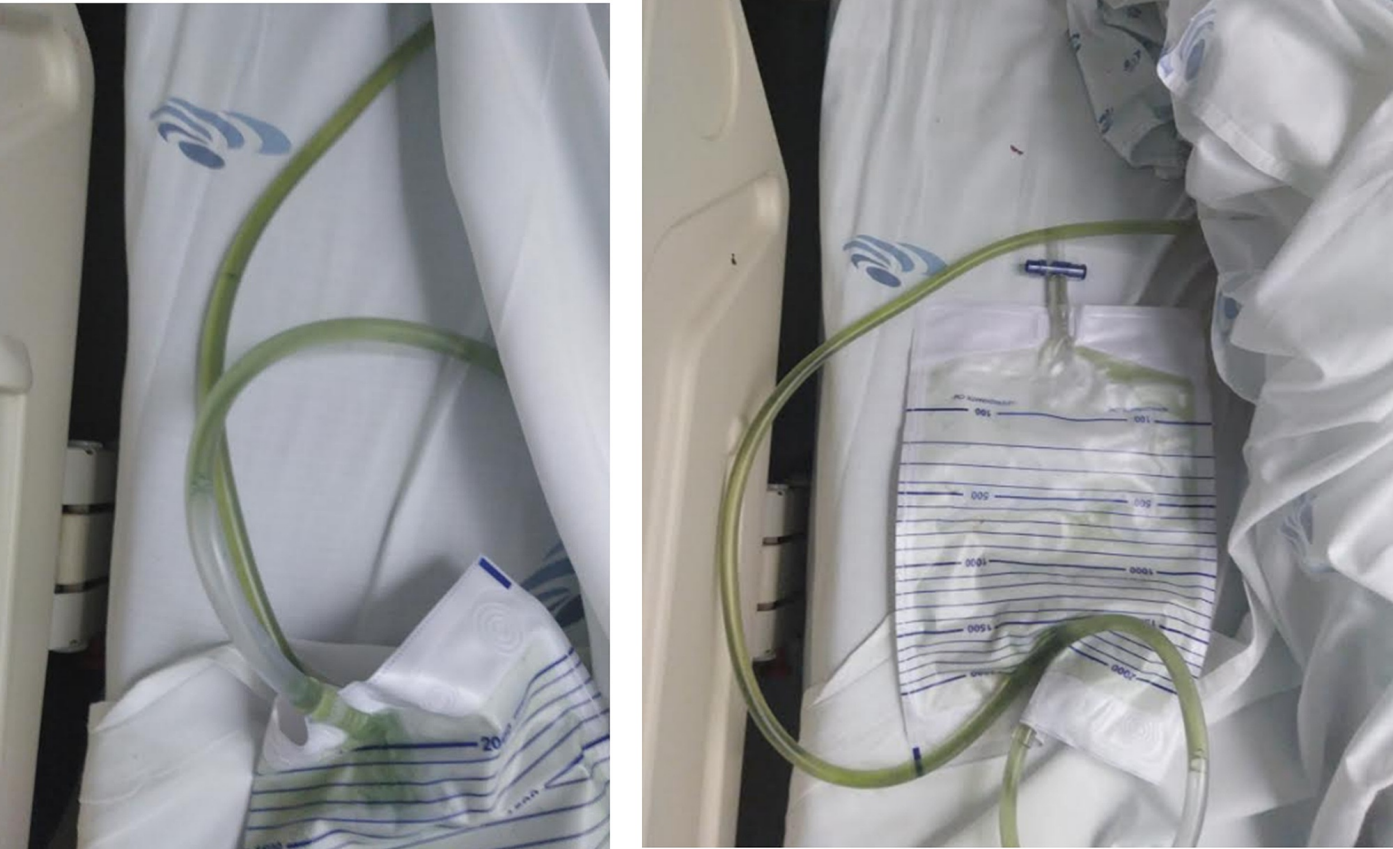
Green-colored urine of the patient

On admission to the ED, the patient was lethargic, with a sparse verbal response. His blood chemistry (Table 1) showed no alterations, and his cerebral computerized axial tomography (CAT) (Figure 1) was unremarkable. His urine, which was collected using vesical catheterization, was green in color and without sediment (Figure 2). The laboratory examination of the urine was unremarkable and was negative for drugs (Table 2).

**Table 1:** Laboratory workup results

Analyte	Result
Hemoglobin	14.7 g/dL
Hematocrit	49.3%
Mean corpuscular volume	88.20 fL
Platelets	237 x 10^3^/µL
Leucocytes	5.63 x 10^3^/µL
Neutrophils	66.80%
Lymphocytes	22.00%
Monocytes	6.90%
Eosinophils	3.50%
Basophils	0.60%
Creatinine	0.84 mg/dL
Urea	38 mg/dL
Sodium	141 mEq/L
Potassium	4.3 mEq/L
C-reactive protein	<0.10 mg/dL
Lactate dehydrogenase	182 U/L
Alanine aminotransferase	33 U/L
Aspartate aminotransferase	28 U/L
Total bilirubin	0,92 mg/dL
International normalized ratio of prothrombin time	1.1
Activated partial thromboplastin time	37 s

**Table 2:** Urine drug screening

Drug	Result
Amphetamines	Negative
Methamphetamines	Negative
Barbiturates	Negative
Benzodiazepines	Negative
Cocaine	Negative
Methadone	Negative
Opioids	Negative
Cannabinoids	Negative
Trycyclic antidepressants	Negative

The clinical team discussed the case with the National Anti-Poisoning Center and received information that this alteration, namely, the change in the normal color of urine, was plausible in cases of flupirtine intoxication (although with no certainty). The patient was then observed by the Neurology Department. After a careful literature search, a similar case of greenish urine following flupirtine intoxication was found in the literature [5].

He was monitored for the next 24 hours in the ED. Gradually, he became more reactive, and his urine lost some of its greenish color. At the time of discharge from the ED to a psychiatric unit, he was aware, although depressed, and showed no other clinical alterations. A follow-up check-up showed no permanent deficits.

## Discussion

Flupirtine is a potent non-opioid analgesic, which is useful in many cases of moderate-to-severe acute pain. Dependency was only reported in two individuals, and no negative psychological or motor function effects were described. It was also studied as a possible novel treatment for some neurological diseases, namely, Creutzfeldt-Jakob disease, Alzheimer’s disease, and multiple sclerosis [6,7].

However, the prevalence of severe hepatotoxicity could not be ignored, and since 2018, its distribution is prohibited in the European Union. Since only two years have lapsed since 2018, the probability of still viable flupirtine tablets in a common household is remarkably high. Clinicians should be aware of its effects, namely, liver toxicity; although the main reported side effects of flupirtine are dose-dependent, hepatotoxicity is not one of them (Table 3).

**Table 3:** Side effects of flupirtine

Side Effects	Prevalence
Ataxia	Not described
Disturbed sleep	Not described
Dizziness	11%
Drowsiness	9%
Dry mouth	5%
Encephalopathy	Not described
Fainting	Not described
Fatigue	Not described
Gastric fullness	5%
Green urine	Not described
Headache	Not described
Heartburn	Not described
Hepatitis	Not described
Increased transaminases levels	Not described
Mood elevation	Not described
Muscle tremor	2%
Nausea	Not described
Nervousness	Not described
Pruritus	9%
Restlessness	Not described
Sedation	Not described
Vomit	Not described

The alteration of the normal color of urine to green, as described in this case, immediately led to a comparison to The Incredible Hulk, the green superhero of Marvel Comics. Greenish urine is not a pathognomonic side effect of flupirtine and has been described in cases of consumption or intoxication by some drugs and dyes; it is also a characteristic of some diseases (Table 4) [8-11]. In the Middle Ages, greenish urine was associated with a choleric personality, and in some traditional medicines, namely Persian, it is associated with the dominance of coldness in the body as a result of humoral imbalance and fluid depletion [8,12].

**Table 4:** Etiology of green urine

Green Urine	
Diseases/medical conditions	Biliverdin; Hartnup disease; familial indicanuria; meconium aspiration; Pseudomonas infection
Drugs	Amitripyiline; azuresin; bromoform; chlorophyll-containing breath mints; cimetidine; clioquinol; flupirtine; flutamide; guaiacol; indomethacin; iodochlorhydroxyquin; magnesium salicylate; methocarbamol; mitoxantrone; phenylbutazone; phenyl salicylate; promethazine (intravenous); propofol; resorcinol; tetrahydronaphthalene; thymol; tolonium; triamterene; zaleplon
Dyes	Diagnex blue; Evans blue; FD&C Blue No. 1; indigo blue; methylene blue; toluidine blue
Poisons	Carbamate; imazosulfuron; mefenacet; phenol
Others	Asparagus; mouthwash overuse

## Conclusions

Irrespective of its potency as a non-opioid analgesic, the prevalence of problematic side effects was higher than its benefits. Although not available on the European market anymore, flupirtine is still sold in some Asian countries and could be entering the European Union at any moment. Clinicians must be aware of typical reactions to this drug.
